# Botulinum toxin-A injection into the anterior belly of the digastric muscle for the prevention of post-operative open bite in class II malocclusions: a case report and literature review

**DOI:** 10.1186/s40902-019-0201-9

**Published:** 2019-04-26

**Authors:** Yei-Jin Kang, Bong Kuen Cha, Dong Soon Choi, In San Jang, Seong-Gon Kim

**Affiliations:** 1 Department of Oral and Maxillofacial Surgery, College of Dentistry, Gangneung-Wonju National University, Gangneung, 28644 Republic of Korea; 2 Department of Orthodontics, College of Dentistry, Gangneung-Wonju National University, Gangneung, 28644 Republic of Korea

**Keywords:** Botulinum toxin-A, Open bite, Malocclusion, Orthognathic surgery, Relapse

## Abstract

**Background:**

Class II malocclusion patients with hyperdivergent facial types are characterized by short mandibular body lengths and anterior open bite. Accordingly, the treatment for hyperdivergent skeletal class II malocclusion is a lengthening of the mandibular body length and a counterclockwise rotation of the mandible. To prevent post-operative relapse, botulinum toxin-A (BTX-A) injection can be a retention modality.

**Case presentation:**

A class II open-bite patient received BTX-A injection to the anterior belly of her digastric muscle for the prevention of post-operative relapse. The relapse was evaluated via a clinical examination and a lateral cephalometric radiograph after the completion of post-surgical orthodontic treatment. The patient showed stable occlusion without any signs of relapse at 15 months post-operatively.

**Conclusion:**

In this case presentation, a single injection into the anterior belly of the digastric muscle was sufficient for the prevention of post-operative open bite.

## Background

A skeletal class II malocclusion has a short mandibular body length relative to the maxilla [1]. Accordingly, the treatment for a skeletal class II malocclusion is a lengthening of the mandibular body via ramus osteotomy. If patients have anterior open bites due to hyperdivergent facial skeletal types, a counterclockwise rotation of the mandible is also required. These types of patients have shown high rates of post-operative relapse and reduced overbite [2]. Many treatment protocols have been introduced to prevent this type of post-operative relapse.

Rigid fixation and suprahyoid myotomy are types of treatment protocols [3, 4]. Inverted L-shaped bicortical screw fixation is considered a reliable fixation method for patients with potential relapse occurrence [5, 6]. Suprahyoid myotomy reduces muscle power that may pull the mandible downward [3]. Although suprahyoid myotomy has shown an acceptable level of success for the prevention of post-operative open bite, it is not widely used at present because of the risk of post-operative morbidity [7].

Botulinum toxin (BTX) originates from bacteria. There are several types of BTX [8]. Among them, BTX-A is the most widely used in clinical practice [9]. In the field of oral and maxillofacial plastic and reconstructive surgery, BTX-A injection is used for the treatment of temporomandibular disorder [10] and for the correction of post-traumatic open bite [11]. In case of open-bite correction, 20 units of BTX-A was injected into the anterior belly of the digastric muscle [11]. The effect of BTX-A injection occurs immediately as a decrease in muscle activity [12]. Then the muscle volume decreases; this usually lasts for 6 months after BTX-A is injected into the masseter muscle [13]. The greatest amount of post-operative relapse after orthognathic surgery appears within 6 months post-operatively [14]. A single injection of BTX-A into the target muscle at the time of surgery may be sufficient to prevent post-operative relapse.

In this case presentation, a class II open-bite patient received BTX-A injection to the anterior belly of her digastric muscle for the prevention of post-operative relapse. Post-operative follow-up continued for 15 months. There was no evident relapse in this patient.

## Case presentation

A 21-year-old female patient was referred from the department of orthodontics to our clinic for orthognathic surgery after the completion of pre-surgical orthodontic treatment. Clinically, she showed anterior open bite with Angle’s class II molar relationship. She also showed hyperplasia of the maxilla and excessive exposure of the maxillary anterior teeth at rest. Her pre-operative radiographs showed a short mandibular body length with a small SNB angle and slight maxillary canting. Her medical history was unremarkable.

**Fig. 1:**
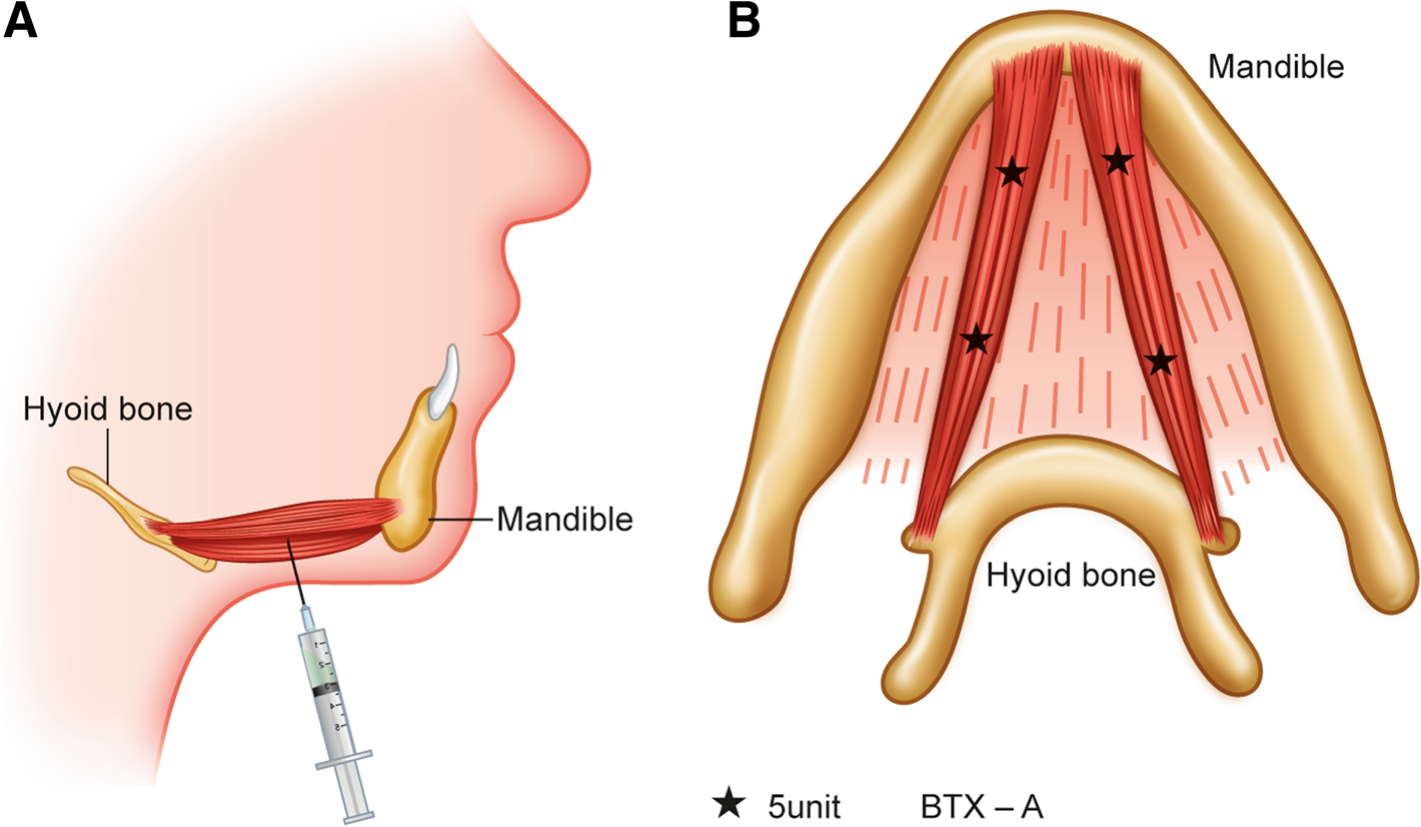
A schematic illustration of the procedure. **a** Injection into the anterior belly of the digastric muscle was performed using a submental approach. **b** The injection points are shown. Each point received 5 units of BTX-A

The patient underwent orthognathic surgery under general anesthesia. For the maxilla, 2 mm of total impaction with an additional 2 mm of posterior impaction, and canting correction was performed using LeFort I osteotomy. For the mandible, 2 mm advancement with a counterclockwise rotation was performed to close the patient’s anterior open bite and establish proper occlusion according to the maxillary movement. After these procedures, genioplasty was performed to establish the patient’s esthetic facial contour. As the required advancement amount of genioplasty was large (8 mm), a double genioplasty was performed. After all of the surgical procedures, 20 units of botulinum toxin (Meditoxin Type A, Medytox, Seoul, Korea) was injected into the anterior belly of the patient’s digastric muscle using a 1-cc syringe immediately after surgery (Fig. 1).

**Fig. 2:**
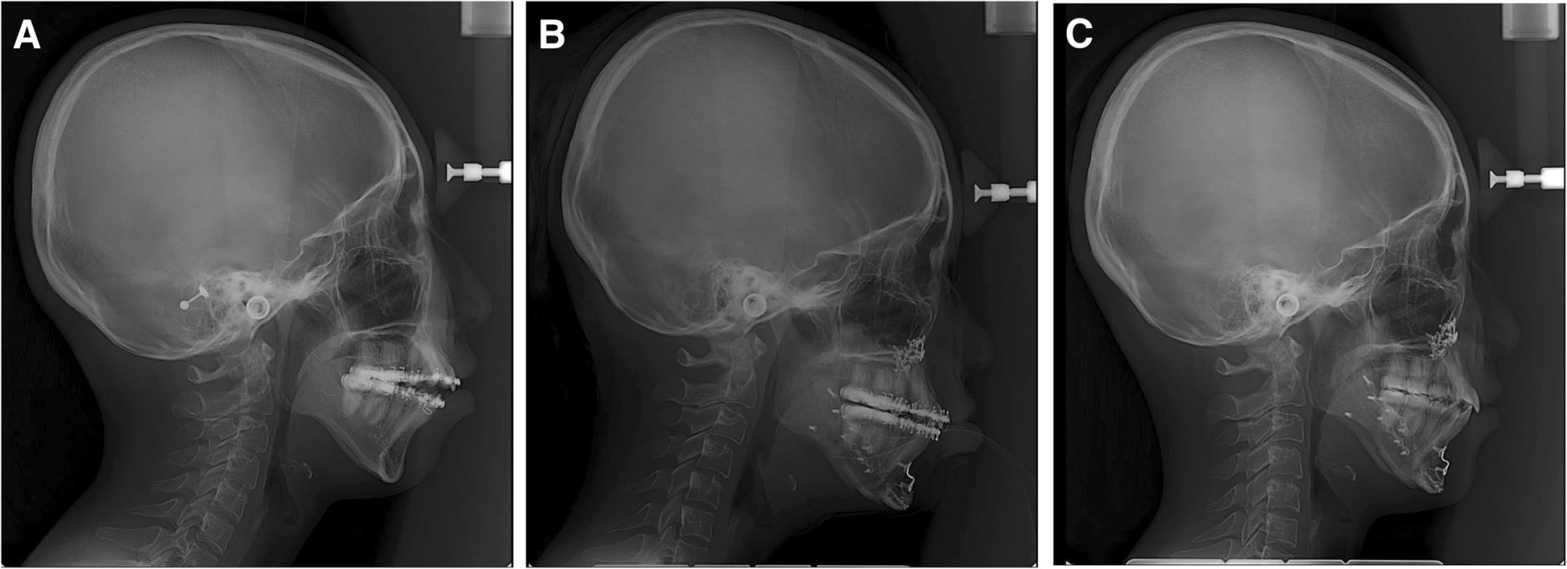
Lateral cephalograms before surgery (**a**), immediately after surgery (**b**), and 15 months after surgery (**c**). In this case, BTX-A injection into the anterior belly of the digastric muscle seemed to prevent post-operative open bite. The patient’s overbite was successfully maintained for 15 months post-operatively. The SNB angle and mandibular plane angle were also stable post-operatively

Relapse was evaluated via a clinical examination and a lateral cephalometric radiograph after the completion of the post-surgical orthodontic treatment. The patient’s overbite was 1.9 mm immediately after surgery and 3.2 mm 15 months post-operatively (Table 1). Her overjet was 3.9 mm immediately after surgery and 3.7 mm 15 months post-operatively (Table 1). The patient showed stable occlusion without any signs of relapse (Fig. 2) and was satisfied with the esthetic results.

**Table 1:** Summary of the patient’s cephalometric measurements

Measurement	Pre-operative	Immediate	15 months later
1. Sagittal relation			
SNA (deg)	75.0	77.6	75.9
SNB (deg)	67.3	73.7	70.7
ANB (deg)	7.7	2.4	4.7
Mandibular length (mm)	109.2	116.9	117.3
Midfacial length (mm)	82.9	88.2	86.4
2. Vertical relation			
Mandibular plane (deg)	47.3	35.7	41.3
Occlusal plane SN (deg)	28.9	26.0	28.9
Palatal plane angle (deg)	7.2	8.0	7.3
Gonial angle (deg)	128.9	129.2	128.3
Lower anterior facial height (mm)	81.3	76.2	76.4
Y axis (deg)	70.1	58.5	62.1
3. Dental relation			
Incisor overbite (mm)	− 2.4	1.9	3.2
Incisor overjet (mm)	7.8	3.9	3.7
4. Soft T. relation			
Nasolabial angle (deg)	99.1	108.8	93.5
Upper lip to E-line (mm)	2.3	−2.1	−0.6
Lower lip to E-line (mm)	6.8	11.5	4.6

## Discussion

Hyperdivergent skeletal class II malocclusion with anterior open bite is challenging because of the high occurrence of post-operative relapse [2]. Accordingly, this was an indication for BTX-A therapy. In this case, BTX-A injection into the anterior belly of the digastric muscle seemed to prevent post-operative open bite. The patient’s overbite was successfully maintained for 15 months post-operatively. The SNB angle and mandibular plane angle were also stable post-operatively (Fig. 2). Considering that BTX-A injection was simple and the procedure has a low rate of complications, BTX-A injection into the anterior belly of the digastric muscle may be considered an additional procedure for the prevention of post-operative anterior open bite [15].

As the mandible is a floating bone suspended by the peroral muscle group, the relative position of the mandible is determined by the balance among the groups [16]. Class II open-bite patients show a small volume of mouth closing muscles and well-developed suprahyoid muscle groups [17]. Ramus surgery for the counterclockwise rotation of the mandible increases the tension on the suprahyoid muscle groups [18]. This tension is considered a major etiologic factor for post-operative relapse [18, 19]. The relapse rate of class II open bite after orthognathic surgery varies considerably from 1.5 to 42.9% [20–24]. Actual amount of relapse may depend on the orthodontic treatment, fixation method, intermaxillary fixation period, osteotomy design, and additional therapy such as myotomy [3–6]. Some procedures such as the fixation method and the intermaxillary fixation period are designed to resist muscle power [4–6]. Posterior impaction of LeFort I osteotomy during surgery reduces the amount of mandibular counterclockwise rotation [25]. As relapse is associated with the correction amount, reduced counterclockwise rotation may be helpful for preventing post-operative relapse [25].

Procedures such as myotomy are designed to reduce muscle power [3]. The rationale for myotomy is similar to BTX-A injection. According to animal study, suprahyoid myotomy group shows less skeletal relapse compared to non myotomy group at 2 years post-operatively [26]. In this paper, pulling action of the suprahyoid musculature is a major risk causing factor in class II open-bite patients [26]. However, a multi-institutional study of 87 individuals did not prove the effectiveness of the suprahyoid myotomy in preventing skeletal relapse [27].

As a complication, anterior open bite has been frequently observed after bilateral mandibular angle fracture [28]. Bilateral mandibular angle fractures result in discontinuity between the mouth opening muscles and the mouth closing muscles [11]. The muscles responsible for opening the mouth are mainly attached to the mandibular anterior region and those for closing the mouth to the mandibular ramus [11]. Accordingly, the influence of the opening muscles is dominant in the mandibular anterior area [11]. BTX-A injection into the anterior belly of the digastric muscle could treat patients with anterior open bite after an open reduction in the bilateral mandibular angle fractures [11]. Radiofrequency therapy for the correction of post-traumatic open bite has similar mechanisms to BTX-A injection [28].

Based on these observations, BTX-A injection into the anterior belly of the digastric muscle was used for the positional stability of a class II open-bite patient who received orthognathic surgery. This study has limitations. First, this was a single case observation. For the stable occlusion after surgery, the importance of post-operative orthodontic treatment should not be ignored. The effect of a BTX-A single injection was difficult to quantify because of other contributing factors. Accordingly, a large-scale prospective study should be conducted for definite conclusions. In addition, there was no experimental support for this protocol. In order to embody the preciseness, the study should be designed and relevant data should be given to prove experimental conclusions. Second, the toxin dosage is very important to avoid any potential complications [29]. The optimal dosage should be tailored using follow-up research. However, BTX-A injection into the anterior belly of the digastric muscle is relatively safe and inexpensive compared to suprahyoid myotomy [15].

## Conclusions

This single case presentation demonstrated that BTX-A injection into the anterior belly of the digastric muscle was used for the positional stability of a class II open-bite patient who received orthognathic surgery. If the correct procedure is performed without abuse, BTX-A injection can be a primary option for the prevention of post-operative relapse.

## Abbreviation

BTX: Botulinum toxin
